# Supplementary material for: Glioma Cell Migration Dynamics in Brain Tissue Assessed by Multimodal Optical Imaging
Source: Biophys J. 2019 Aug 15;117(7):1179–88. doi: 10.1016/j.bpj.2019.08.010 (PMC6818150; doi:10.1016/j.bpj.2019.08.010)
Supplement: Document S1. Figs. S1–S4 [file mmc1.pdf]

Biophysical Journal, Volume *117*

Supplemental Information

Glioma Cell Migration Dynamics in Brain Tissue Assessed by Multimodal Optical Imaging

Chao J. Liu, Ghaidan A. Shamsan, Taner Akkin, and David J. Odde

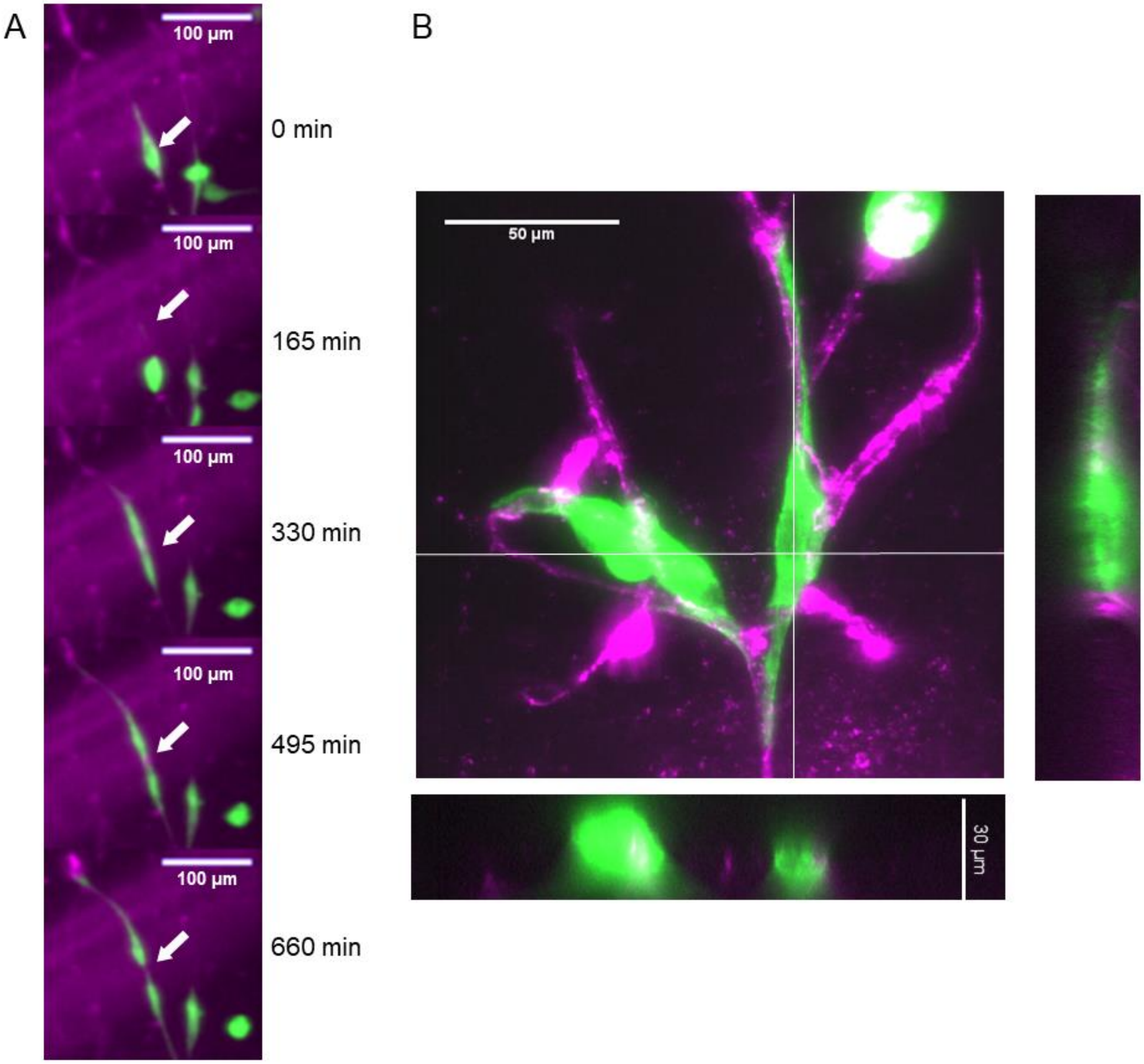

Supplementary Fig. 1 U251 cell migration dynamics in normal mouse brain slices.

(A) U251 cell division occurs in the perivascular space (indicated by the white arrows).
(B) Orthogonal views of perivascular U251 cell invasion in brain slice.

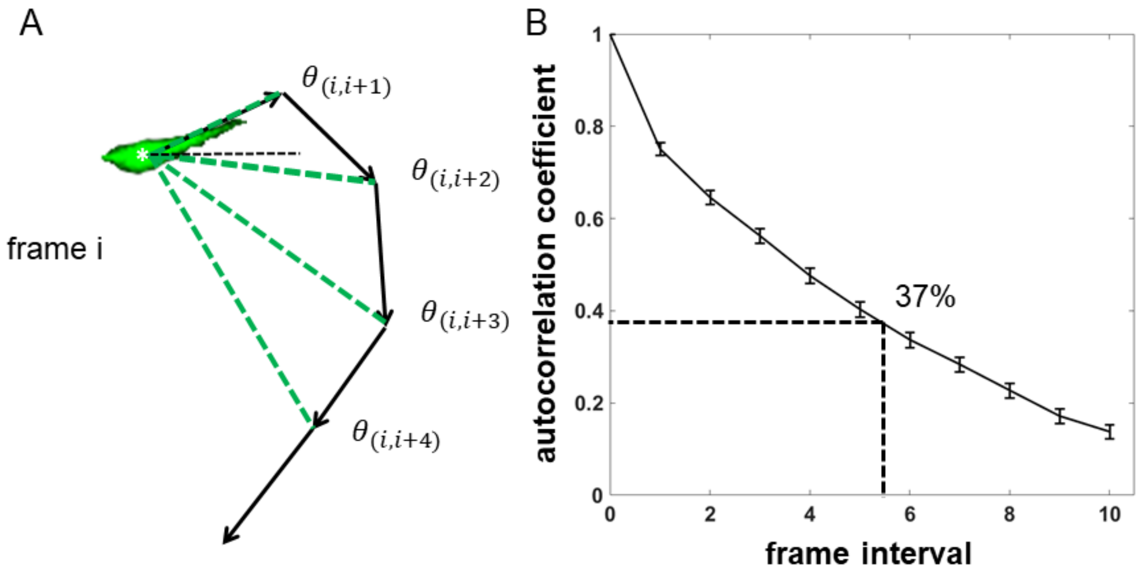

Supplementary Fig. 2. Calculation of cell migration angle

(A) Calculation of cell migration angles over different time intervals. (B) The autocorrelation coefficient of migration angles as a function of frame intervals (15 min/frame). The decay to 0.37 reflects the 1/e decay time. Measurements separated by 6 intervals were considered statistically uncorrelated.

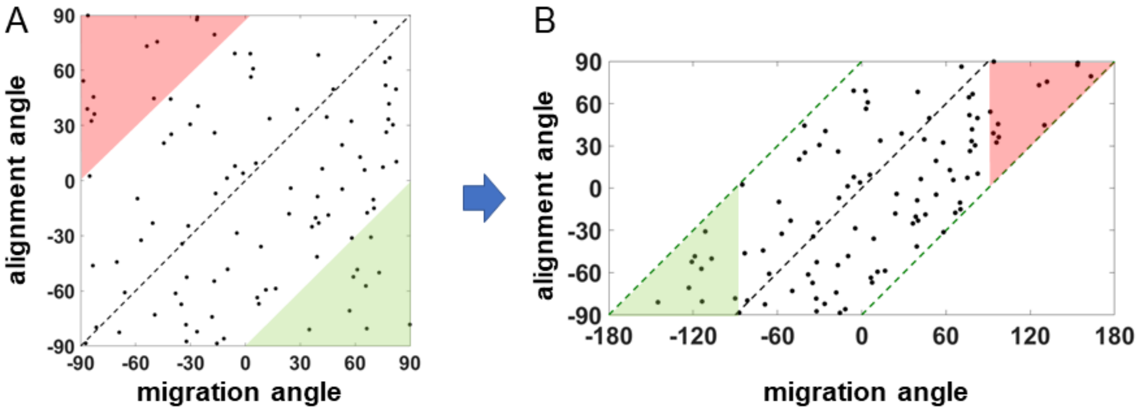

Supplementary Fig. 3 The analysis on the alignment of cell migration and local structure

100 simulated random walkers cells in the two shaded areas of Supplementary Fig. 3A are translated to the parallelogrammatic coordinate system (Supplementary Fig. 3B) separately. Supplementary Fig. 3B is used for the alignment index calculation of Fig. 3A.

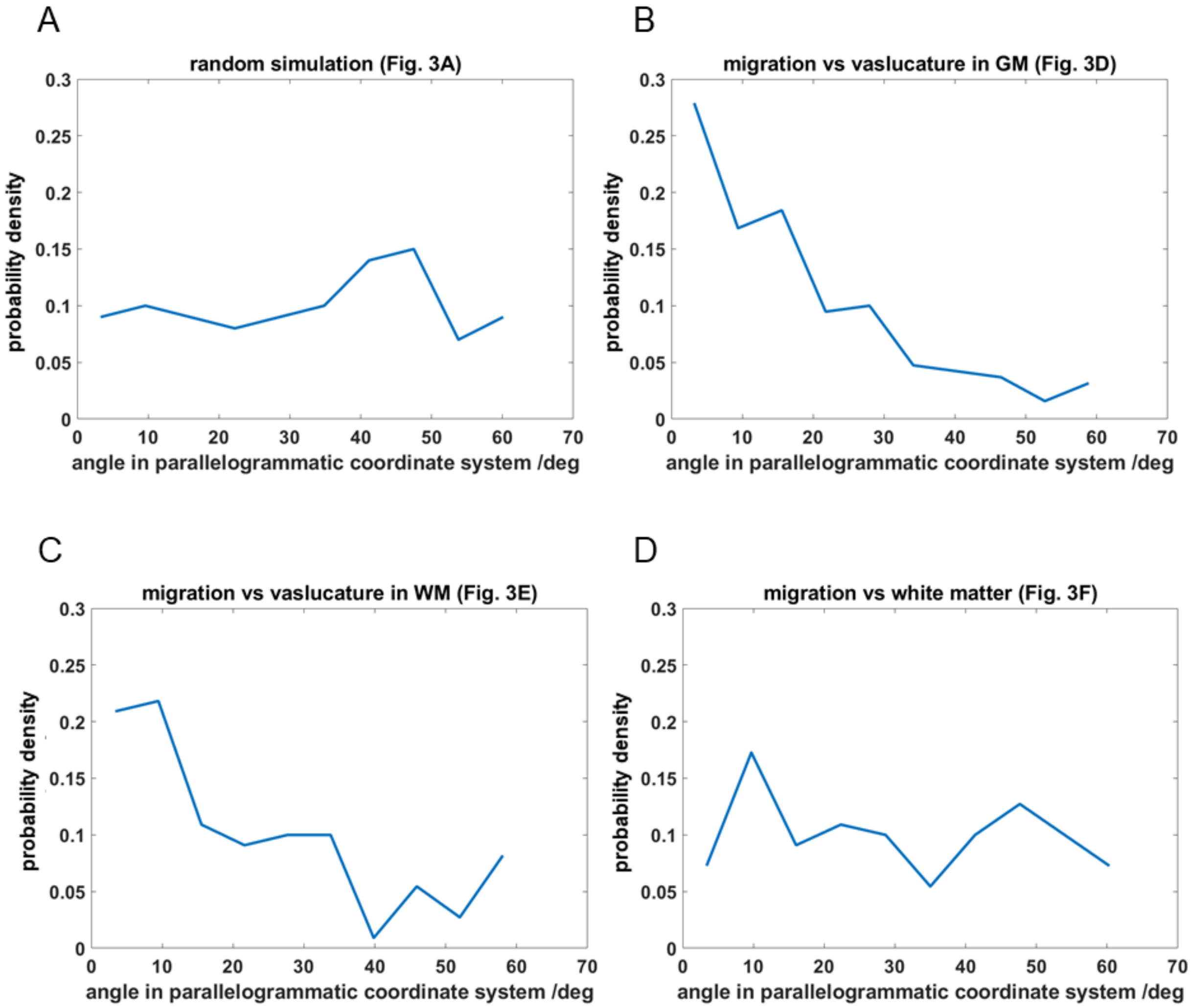

Supplementary Fig. 4 The probability density function of the scatter plots in Fig. 3

(A) The simulated $\phi = 0$ case. Cell migration angles are plotted versus the vessel orientations for cells in the gray matter (B) and white matter (C). (D) Cell migration angles are plotted against white matter tract orientations for cells in the white matter.

Supplementary videos

Supplementary video 1 (corresponding to Fig. 2A and 2B):

Cell migration along vasculature and the computational orientation maps.

Supplementary video 2 (corresponding to Fig. 2C and 2D):

Cell migration along white matter tracts and the computational orientation maps.

Supplementary video 3 (corresponding to Fig. 5A):

U251 cell deforms local vasculature during migration (vessel was pulled towards the cell at the leading edge).

Supplementary video 4 (corresponding to Fig. 5D):

U251 cell deforms local vasculature during migration (vessel was pushed away from the cell at the leading edge).
