# Supplementary material for: Glioma Cell Migration Dynamics in Brain Tissue Assessed by Multimodal Optical Imaging
Source: Biophys J. 2019 Aug 15;117(7):1179–88. doi: 10.1016/j.bpj.2019.08.010 (PMC6818150; doi:10.1016/j.bpj.2019.08.010)
Supplement: Document S2. Article plus Supporting Material [file mmc6.pdf]

Biophysical Journal
Article

Biophysical Society

Glioma Cell Migration Dynamics in Brain Tissue Assessed by Multimodal Optical Imaging

Chao J. Liu,[1] Ghaidan A. Shamsan,[1] Taner Akkin,[1] and David J. Odde[1,*]
[1]Department of Biomedical Engineering, University of Minnesota, Minneapolis, Minnesota

ABSTRACT Glioblastoma is a primary malignant brain tumor characterized by highly infiltrative glioma cells. Vasculature and white matter tracts are considered to be the preferred and fastest routes for glioma invasion through brain tissue. In this study, we systematically quantified the routes and motility of the U251 human glioblastoma cell line in mouse brain slices by multimodal imaging. Specifically, we used polarization-sensitive optical coherence tomography to delineate nerve fiber tracts while confocal fluorescence microscopy was used to image cell migration and brain vasculature. Somewhat surprisingly, we found that in mouse brain slices, U251 glioma cells do not follow white matter tracts but rather preferentially migrate along vasculature in both gray and white matter. In addition, U251 cell motility is ~2-fold higher in gray matter than in white matter (91 vs. 43 $\mu m^2/h$), with a substantial fraction (44%) of cells in both regions invading without close association with vasculature. Interestingly, within both regions, the rates of migration for the perivascular and televascular routes of invasion were indistinguishable. Furthermore, by imaging of local vasculature deformation dynamics during cell migration, we found that U251 cells are capable of exerting traction forces that locally pull on their environment, suggesting the applicability of a "motor-clutch"-based model for migration in vivo. Overall, by quantitatively analyzing the migration dynamics along the diverse pathways followed by invading U251 glioma cells as observed by our multimodal imaging approach, our studies suggest that effective antiinvasive strategies will need to simultaneously limit parallel routes of both perivascular and televascular invasion through both gray and white matter.

SIGNIFICANCE Which biophysical routes do brain cancer cells use to invade into brain tissue? Whereas previous studies have reported glioma cells in proximity to capillary and axonal white matter tracts, the actual dynamic analysis of cell motion has been limited. Using a multimodal imaging approach on human U251 glioma cell migration into mouse brain slices ex vivo, we found little correlation between white-matter-tract alignment and glioma cell migration. Rather, cells frequently associate with blood vessels. However, the ability to migrate in the perivascular space had no effect on cell migration relative to televascular invading cells, highlighting the importance of measuring dynamics to infer invasion rather than relying on static images as has been the practice in the field.

INTRODUCTION

Glioblastoma is an aggressive malignant primary brain tumor with dismal prognosis that is characterized by highly infiltrative migration of cancer cells into the healthy brain (1). The mechanism and the physical paths to invade healthy brain have been extensively studied by pathologists and neurosurgeons for decades. In 1938, Hans Scherer used postmortem histological analysis to conclude that gliomas migrate along existing brain structures (2). These structures, later named Scherer's structures, include the brain parenchyma, pre-existing blood vessels, white matter tracts, and the subarachnoid space below the meningeal covering of the brain (3).

Among the Scherer's structures, vasculature and white matter tracts account for the primary structural alignments in the brain. The current interpretation is that these are therefore the preferred and fastest routes for glioma invasion (1,2). Blood vessels are considered to play an important role in glioma migration because dynamic imaging studies provided ample evidence that gliomas cells frequently utilize blood vessels as guides to migrate in brain tissue (4–8). At the same time, white matter tracts are also considered to be important for glioma migration (2,3,9). In particular, the spreading of glioblastoma from one hemisphere to the other is thought to result from invasion via white matter tracts connecting the two lobes, such as the corpus callosum or anterior commissure (10,11). The presence of glioma cells along

Submitted March 13, 2019, and accepted for publication August 12, 2019.
*Correspondence: oddex002@umn.edu
Editor: Cynthia Reinhart-King.
https://doi.org/10.1016/j.bpj.2019.08.010
© 2019 Biophysical Society.
This is an open access article under the CC BY license (http://creativecommons.org/licenses/by/4.0/).

white matter tracts was also observed in rat xenografts by histological images (12,13). These previous studies typically drew conclusions from magnetic resonance imaging or histological approaches, i.e., via nondynamic imaging. However, the colocalization of glioma cells with white matter tracts from such static data does not necessarily imply that white matter tracts are a major migratory pathway. Rather, it is formally possible that these regions actually have slower migration, and as a result, there is accumulation as observed in the static images. Moreover, it is unclear whether white matter induces glioma cells to migrate faster. For example, Giese et al. measured the migration rate of several glioma cell lines on glass substrates coated with myelin extracted from human brain samples and discovered that none of the cell lines studied showed higher migration rates compared with merosin (α2 chain of laminin-2)-coated substrates (14). Although intriguing, such in vitro studies point to the importance of quantifying cell motility dynamically in the native brain microenvironment. However, exogenous dyes for in vivo labeling of white matter tracts usually introduce toxicity, which limits the acquisition of dynamic data (15). Thus, there is a need for new label-free imaging techniques to delineate white matter tracts and correlate them with glioma cell migration dynamics.

Among such label-free techniques, polarization-sensitive optical coherence tomography (PS-OCT) provides quantitative contrast that originates from tissue back scattering. PS-OCT is capable of generating depth-resolved images of reflectivity, phase retardance, and optic axis orientation. Because of the birefringent property of myelin sheath, nerve fiber tracts as small as a few tens of micrometers can be resolved from phase retardance images (16). The in-plane fiber alignments of nerve fibers can also be quantified. PS-OCT, as a label-free method to study the brain, has recently been used to advance our understanding of brain connectivity (17) and neurodegeneration (18).

Organotypic brain-slice culture as a glioma invasion assay was established by Ohnishi et al. (19); it overcomes the fundamental limitations of conventional in vitro two-dimensional (2D) cell culture assays defining specific matrix components that lack the biochemical composition, architecture, and mechanics of local three-dimensional (3D) brain extracellular matrix and stromal microenvironment (13,20–22). In this study, we used the human glioblastoma cell line U251 in mouse brain-slice organotypic culture to investigate glioma cell migration behavior and dynamics by multimodal optical imaging. A swept-field confocal imaging system was used to image U251 cells expressing GFP-actin and brain vasculature stained by isolectin IB4-Alexa Fluor 568 conjugate. In addition, PS-OCT was used as a label-free approach to delineate the birefringent tissue that is characteristic of white matter tracts and registered with the confocal videos. To our knowledge, this is the first study to incorporate white matter tracts together with vasculature for a comparative dynamic analysis of glioma cell migration along the major routes of invasion. Here, we quantitatively analyzed the extent of U251 glioma cell migration along white matter tracts in normal mouse brain slices, as well as the extent and speed of perivascular and televascular spaces in both gray and white matter regions as delineated via multimodal optical imaging. Surprisingly, we find that U251 cell migration lacks alignment with white tracts and instead follows both perivascular and televascular routes of invasion in both white and gray matter. We find that a "motor-clutch" model description is consistent with the traction force dynamics in brain tissue.

MATERIALS AND METHODS

U251 cells culture

Human glioma cell line U251 was originally obtained from Dr. G. Yancey Gillespie (University of Alabama at Birmingham) and was authenticated using the short tandem repeat assay (University of Arizona Genetics Core). For the brain-slice studies, stably transfected U251-GFP-actin cells were used (23). Cells were cultured in T25 plastic tissue culture flasks (353108; Becton Dickinson, Franklin Lakes, NJ) in a humidified 37°C, 5% CO_2 incubator. Dulbecco's modified Eagle's medium/F-12 (31765-035; Gibco, Gaithersburg, MD) with 8% fetal bovine serum (10438-026; Gibco) and 1× penicillin/streptomycin (30-001-CI; Corning, Corning, NY) was used to culture the cells. Before plating cells onto brain slices for migration studies, cells were removed from the flask using 0.25% trypsin with EDTA in Hanks' balanced salt solution (25-052-CI; Gibco).

Preparation of mouse brain slices

All animal treatments and experiments are in accordance with Institutional Animal Care and Use Committee at the University of Minnesota approved protocols. Adult friend virus B mice were terminally anesthetized in a CO_2 chamber and then perfused transcardially with isotonic saline. The brains were extracted and transferred into chilled oxygenated artificial cerebrospinal fluid (124 mM NaCl, 2.5 mM KCl, 2.0 mM $MgSO_4$, 1.25 mM KH_2PO_4, 26 mM $NaHCO_3$, 10 mM glucose). The cerebrum was sectioned into 300 μm coronal slices using a vibratome (VT1000; Leica Biosystems, Richmond, IL). Typically, the slice near Bregma −1.5 mm was chosen for the next steps. The experiment was repeated five times.

PS-OCT imaging of brain slices

Fig. 1 *A* explains the experimental procedures for the mounting of brain slices in preparation for multimodality imaging. One coronal slice (half hemisphere) was selected to be imaged by PS-OCT. A custom spectral-domain PS-OCT system was used in this work. Detailed descriptions of the imaging system can be found in earlier publications (24,25). Briefly, a water-immersion microscopic objective (UMPLFLN 10× W; Olympus, Tokyo, Japan) ensures a lateral resolution of 4 μm with a 1.25 × 1.25 mm field of view (26). The system provides an axial resolution of 5.5 μm. The whole sample was imaged by tile-scan in the lateral plane through computer-controlled actuators. PS-OCT acquires 3D data of multiple contrasts, including reflectivity, retardance, and optic axis orientation. Retardance contrast, which highlights white matter tracts, was used to generate an *en face* image by averaging to a depth of ~70 μm to match the fluorescence depth of imaging and typical cell invasion depth in tissue. The PS-OCT imaging with necessary preparation typically took ~30 min.

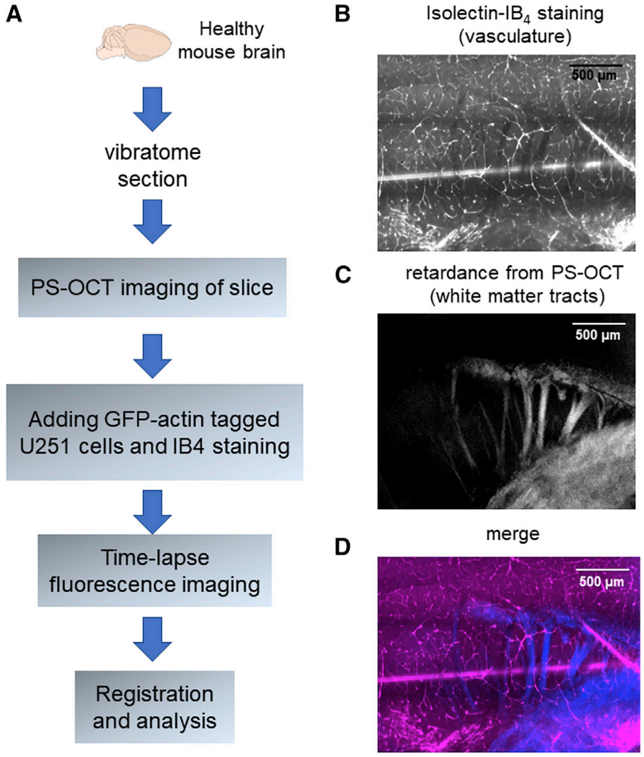

FIGURE 1 Experimental procedures and multimodal registration. (*A*) Experimental procedure schematic and workflow are shown. (*B*) Isolectin IB4 fluorescent staining and (*C*) retardance contrast from PS-OCT are registered in the composite image (*D*). Scale bars: 500 μm. To see this figure in color, go online.

Organotypic brain-slice coculture with glioma cells

After performing PS-OCT imaging, the brain slice was transferred onto No. 0 glass-bottom 35 mm culture dishes (P35G-0-20-C; MatTek, Ashland, MA). U251 cells (500,000–800,000) were plated onto the brain slice. Isolectin GS-IB4 (Alexa Fluor 568; Molecular Probes, Eugene, OR) was added to the brain slice to label the vasculature. Isolectin IB4 labels endothelial cells and has been used as a robust marker for vasculature (27). The cells were cocultured with the brain slice for 4 h before imaging to allow for cell infiltration into the brain slice. Cells typically invade up to 50 μm into the tissue. Before imaging, the brain slice was washed several times using cell culture media. A tissue culture anchor (SHD 42-15; Warner Instruments, Hamden, CT) was placed on top to prevent tissue drift during imaging.

Live-cell confocal fluorescence time-lapse imaging and analysis of cell migration

For experiments on brain slices, the slice was imaged on a Zeiss LSM 7 Live swept-field laser confocal microscope (Carl Zeiss, Oberkochen, Germany) with a 10× 0.45 NA objective lens capable of simultaneous imaging in both the green (U251-GFP-actin) and red (IB4) channels as previously described (22). Maximal intensity projections from multiple z-stacks were used to generate 2D images for quantitative shape and motion analysis. The number of z-stacks was adjusted to ensure that the data acquisition of the whole slice was completed under 15 min (six to eight planes with 10 μm separation were typically used). The z-stacks were then imaged every 15 min for up to 16 h at 37°C in a humidified 5% CO_2 environment. We observed U251 cell division events in this assay. The montage in Fig. S1 *A* shows a cell dividing in the perivascular space.

By using a 40× 0.95 NA objective, Fig. S1 *B* shows the orthogonal views of U251 cell invasion in the brain slice. Time-lapse imaging at 40× magnification was used to show the dynamics of local vasculature during cell migration. Single cells with clear direction of migration in the field of view were imaged at 20 s intervals. The deformation rate of the blood vessels was quantified by FlowTrack v2.0 as of March 2019, which is available for download from oddelab.umn.edu (28).

Single cell migration was tracked by a custom-written image segmentation algorithm (23). The cell region was separated from the image and fitted with an ellipsoid. The centroid coordinates (x_i, y_i), areas and major and minor axis lengths of cells were tracked for each frame i. The random motility coefficient (D) for the projected 2D cell migration was calculated by linear fitting to the mean-squared displacement (MSD) versus time (t) as shown in Eq. 1.

$$MSD(t) = 4Dt, \tag{1}$$

Cell migration angles were calculated using Eq. 2, where δ represents the frame intervals for this angle calculation (Fig. S2 *A*).

$$\theta(i, i+\delta) = \tan^{-1}\left(\frac{y_{i+\delta} - y_i}{x_{i+\delta} - x_i}\right), \tag{2}$$

The migration angle of a cell θ was considered as the median of the angles calculated across the time series, with angles ranging from −90 to +90°. To define the frame interval for angle calculation, the autocorrelation function of migration angles as a function of time was evaluated (Fig. S2 *B*). The coefficient drops to 1/e at ~80 min ($\delta = 5.5$). Therefore, in the following analyses, we used frame intervals of 60 min ($\delta = 4$) to determine statistically independent cell migration angles.

Registration of multimodal images

Stage drift and tissue relaxation during fluorescence imaging were registered by an affine transformation using ImageJ StackReg plug-in (École Polytechnique Fédérale de Lausanne, Lausanne, Switzerland). The whole slice sample was imaged by both PS-OCT and fluorescence modalities. All contrasts from PS-OCT were registered to the fluorescence images by performing intensity-based registration using isolectin IB4 staining and reflectivity from PS-OCT. The Pearson's correlation coefficient between the two modalities is 0.86 ± 0.02 (mean ± SD, $n = 5$ experiment repeats). Fig. 1 shows an example of the registered images: isolectin IB4 staining channel (Fig. 1 *B*) and retardance contrast from PS-OCT (Fig. 1 *C*) are registered in the composite image (Fig. 1 *D*).

Persistent-random-walk simulations for defining alignment index

A simulation for a persistent random walk was used to define the alignment index to estimate the expected alignment between cell migration orientations and structural orientations that are assumed to be uncorrelated, based on Bergman and Zygourkis (29). An initial alignment orientation ϕ was randomly assigned to a cell. The cell migrates with speed s and persistence P starting from (0, 0). The initial orientation of the cell is set to be $\theta_0 = \phi$. The orientation of the next step was decided by comparing a random generated number r_1 ($0 < r_1 < 1$) with τ/P, where τ is the time interval between steps. The cell changes orientation to $\theta_1 = 2\pi r_2$ when $r_1 < \tau/P$, where r_2 is a new randomly generated number ($0 < r_2 < 1$). Otherwise, the cell keeps the old orientation $\theta_1 = \theta_0$. The displacement of migration in the x and y axes is given as $s\tau\cos\theta_1$ and $s\tau\sin\theta_1$. The iteration continues until the given steps

are finished as the coordinates of each step and the corresponding alignment orientation are recorded.

Stochastic cell migration simulator

The cell migration simulator models an entire cell by mechanically connecting several motor-clutch modules together and balancing the forces at the central node. The simulator has been described previously and well-characterized by publications from our group (22,23,30,31). The parameters used in this study are given in Table 1. To simulate the televascular space, cell migration simulator (CMS) v1.0 was used without modification to simulate cells without restrictions, current as of March 2019, which is available for download from oddelab.umn.edu. The diameter of the vasculature in the mouse brain can range from less than 10 μm to as large as 100 μm for the pial surface vessels (32). To simulate the perivascular space, CMS v1.0 was modified by restricting the cells within 50 μm for one dimension while free in another. We chose 10 pN/nm as the substrate spring constant for both gray and white matter, equivalent to ~10 kPa (28).

Statistical analysis

Distributions obtained for alignment indices and random motility coefficients of cells in different categories were compared using a nonparametric Kruskal-Wallis test. Dunn-Sidak's test was used to counteract the problem of multiple comparisons if more than two groups were involved (22,23). All error bars represent standard error of the mean (SEM).

RESULTS

Imaging of white matter tracts and blood vasculature in normal mouse brain tissue

The multimodal optical imaging approach allows us to assess the relative contributions of vasculature versus white matter tracts. For the former, we used a multiscale vessel filter, which was based on the analysis of the Hessian of the isolectin IB4 images. The filter outputs the likeliness of the perivascular space and correspondingly the vessel orientation (33). The vessel orientation was used to study the alignment between cell migration and vasculature. Fig. 2 *A* shows a perivascular cell as it migrates along a blood vessel. Fig. 2 *B* presents the calculated vessel orientation map superimposed with the cell trajectory.

PS-OCT was used to identify the white matter tracts in the brain tissue slices. Similarly, Fig. 2 *C* shows an example of cell migration along white matter tracts. The retardance contrast in blue illustrates the white matter tracts. Retardance, the degree of angular shift between the orthogonal polarization channels of the incoming light, is a quantitative measure of tissue birefringence. We used structure tensor-based analysis of the *en-face* retardance images to calculate the orientations of the white matter tracts (34). This method assesses the gradients of images in image subregions to generate a matrix whose eigen-decomposition estimates the orientations of white matter tracts. The white matter orientations were used to study the alignment between cell migration and white matter tracts. Fig. 2 *D* presents the calculated white matter orientation map superimposed with the cell trajectory. The brightness of the map corresponds to the retardance and the orientations are indicated by the color wheel.

TABLE 1 Cell Migration Simulator Parameter Values

Symbol	Parameter	Value
N_m	number of motors	1000
N_c	number of clutches	850; 1000
A_{tot}	total possible actin protrusion length	100 μm
V_p*	maximal actin polymerization velocity	200 nm/s
k_{mod}*	maximal module birth rate	1 s^{-1}
k_{cap}	module capping rate	0.001 s^{-1}
I_{in}	initial module length	5 μm
I_{min}	minimal module length	0.1 μm
k_{cell}	cell spring constant	1000 pN/nm
$n_{c,cell}$	number of cell body clutches	10
F_m	motor stall force	2 pN
V_m*	unloaded motor velocity	120 nm/s
k_{on}	clutch on-rate	1 s^{-1}
k_{off}*	clutch unloaded off-rate	0.1 s^{-1}
k_{in}*	maximal number of module motors	100
V_m*	unloaded motor velocity	120 nm/s
n_c*	maximal number of module clutches	$0.1xN_c$
k_c	clutch spring constant	0.8 pN/nm
F_b	characteristic clutch rupture force	2 pN
k_s	substrate spring constant	10 pN/nm

U251 cell migration aligns with vasculature more than white matter tracts

To understand the alignment between cell migration and local structure, we first computationally simulated cell migration paired with alignment angles. Fig. S3 *A* simulates the random case, in which the migration angles of each cell are independent of the alignment direction. Each point in the plot represents one cell. If migration and alignment angle are highly correlated, it indicates the cell is migrating along a local alignment, and thus, the points should lie very near to the diagonal line in this plot. We wrapped the cells in shaded areas to the parallelogrammatic coordinate system (Fig. S3 *B*) from the square system shown in Fig. S3 *A*. Then in Fig. 3 *A*, the distance between each point to the black diagonal line, *d*, can be used as a measure of alignment. We simulated 100 random walkers and calculated the distance for the random case as $d_R = 32°$. We further defined the alignment index (Eq. 3) by normalizing *d* of a cell to d_R and quantified the alignment of migration with local structure,

$$\varphi = 1 - {}^{d}/_{d_R}, \tag{3}$$

where φ denotes the alignment index. By adjusting the persistence of the simulated cell migration, Fig. 3, *B* and *C* show the scatter plots for $\varphi = 0.3$ and $\varphi = 0.7$. The migration becomes more aligned with the structure when the persistence increases. The variability of alignment index serves as a measure of the deviation of cell migration angle from local vessel or tract angle. An alignment index of 1 implies that cell migration is perfectly aligned to local vessels or tracts, whereas an index of −1 implies migration

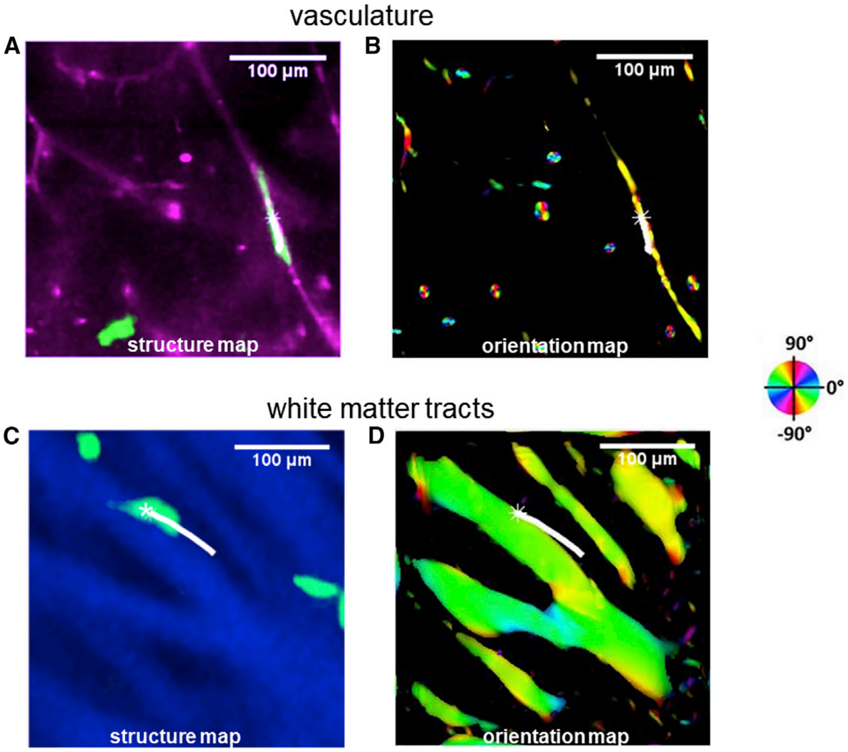

FIGURE 2 Multimodal optical imaging of migrating glioma cells, blood vessels, and white matter tracts. (*A*) A perivascular U251 glioma cell (*green*) imaged by confocal microscopy migrates in the perivascular space, with blood vessels also image by confocal microscopy (*magenta*). (*B*) The calculated vessel orientation map superimposed with the cell trajectories is shown. See Video S1. (*C*) An example of a U251 cell (*green*) migrating along white matter tracts (*blue*) imaged by PS-OCT is given. (*D*) The calculated white matter orientation map superimposed with the cell trajectories is shown. See Video S2. The brightness of the maps is controlled by vessel-likeliness and retardance, separately. The orientations are indicated by the color wheel. Scale bars: 100 μm. To see this figure in color, go online.

perpendicular to local vessels or tracts, and intermediate values closer to 0 imply increasing levels of misalignment from the aligned (+1) or perpendicular (−1) cases.

We examined the alignment between cell migration and vasculature in both gray matter and white matter, which are distinguished by retardance contrast from PS-OCT. We performed the comparison of the alignment index on cell migration alignment with vasculature in the gray (Fig. 3 *D*, $n = 190$) and white matter (Fig. 3 *E*, $n = 110$). In the white matter, blood vessels and white matter tracts were included in this comparison. Fig. 3 *F* shows the cell migration angles as a function of the local white-matter-tract orientations ($n = 110$). In Fig. 3 *G*, no significant difference was found between cell migration alignment with vasculature in the gray and white matter ($p = 0.3$). However, we found higher alignment index between cell migration and vasculature compared with white matter tracts ($p = 0.001$). This suggests that U251 cell migration is more aligned with vasculature rather than white matter tracts in the white matter. At the same time, the alignment between cell migration and white-matter-tract angles shows no statistical difference from the random control (Fig. 3 *A*, $p = 0.99$). Fig. S4 shows the probability density function of the analyzed scatter plots in Fig. 3. Our quantitative analysis confirmed earlier findings that blood vessels are major migration pathways for glioma cells (1,3–8). Our data show that blood vessels are widely used for glioma cells both in gray matter and white matter. Especially in the white matter, blood vessels are also the preferential pathways compared with white matter tracts. It is important to note that in our assay, we also discovered examples of cell migration along white matter tracts (Fig. 2 *C*). However, the frequency of such events was consistent with mere chance. Even in structures like the corpus callosum, which is believed to account for contralateral invasion, glioma cells could still use blood vessels instead of white matter tracts as the aligned structure of choice, consistent with our observations.

U251 cell motility is modestly higher in gray matter than in white matter

Fig. 4 *A* presents the averaged MSDs as a function of time interval of cells tracked in the perivascular and televascular space. After fitting the MSDs with Eq. 1, the perivascular and televascular cells show no difference in terms of random motility coefficient in both gray and white matter regions (Fig. 4 *B*). Similar to cell migration behavior in 3D aligned collagen gels (35), the presence of vascular alignment does not alter cell motility. On the other hand, Fig. 4 *C* presents the averaged MSDs of cells tracked in gray matter and white matter separately. Somewhat surprisingly, we found that the random motility coefficient of cells in gray matter region is $91.4 \pm 10.1\ \mu m^2/h$, which is ~2-fold greater than in white matter, where it is $43.1 \pm 7.1\ \mu m^2/h$ (Fig. 4 *D*).

U251 cells deform local vasculature during migration

To assess the forces underlying cell migration in brain slices, we further visualized the cell traction behaviors in the

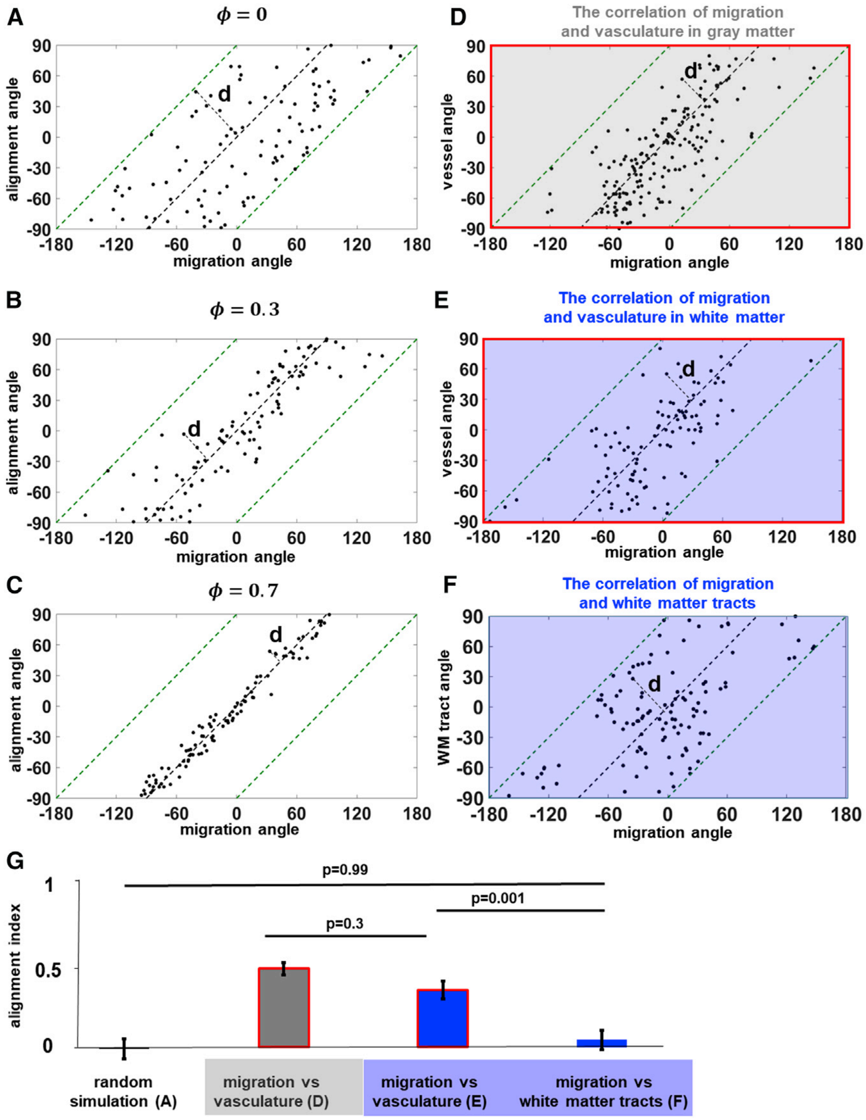

FIGURE 3 In white matter, U251 cells follow vasculature rather than white matter tracts. The scatter plots show the alignment between migration angle and structural angle in simulated cases from random to aligned scenarios ($n = 100$): (*A*) $\phi = 0$, (*B*) $\phi = 0.3$, and (*C*) $\phi = 0.7$. ϕ denotes the alignment index. Cell migration angles are plotted versus the vessel orientations for cells in the gray matter (*D*, $n = 190$) and white matter (*E*, $n = 110$). (*F*) Cell migration angles are plotted against white-matter-tract orientations for cells in the white matter ($n = 110$). (*G*) The analysis of alignment indices indicates U251 cells are equally correlated with vasculature in gray matter and white matter ($p = 0.3$). However, U251 cells tend to follow blood vessels rather than white matter tracts in the white matter ($p = 0.001$). See Fig. S4 for the probability density function of the corresponding scatter plots. Error bars represent SEM. To see this figure in color, go online.

brain-slice migration assay. Fig. 5 *A* shows a U251 cell exerting force on the local blood vessel along which it migrates. The kymograph at the leading edge of cell migration shows that the cell applies load by pulling the blood vessel toward the cell body (Fig. 5 *B*). Analysis on the kymograph shows the vessel was deformed by a rate of 6.7 nm/s. On the other hand, at the trailing edge where the cell is losing contact with the environment, the blood vessel relaxes away from the cell (Fig. 5 *C*). The pulling forces at the leading edge and relaxation dynamics at the trailing edge are both consistent with the motor-clutch mechanism for cell migration because molecular clutches link myosin-driven F-actin motion to the substrate to load the environment at the leading edge and detach from it at the trailing edge (28). Unlike the motor-clutch model, the osmotic engine model utilizes directed water permeation instead of F-actin dynamics to mediate propulsion, which drives cell migration in 3-μm-wide polydimethylsiloxane microchannels for some cell types (36). In contrast to the motor-clutch model, the osmotic engine model predicts the opposite force dynamics at the leading edge, where the cell should push away the blood vessel because of the osmotic pressure. We also observed U251 cells occasionally pushing the local blood vessel along which it migrates, as shown in Fig. 5 *D*. The kymograph in Fig. 5 *E* at the leading edge shows that the cell deformed the vessel at a rate of 2.3 nm/s. The majority of cells (six out of eight observed cells with clear direction of migration) were observed to exert pulling forces at the leading edge, deforming the vessels at an average rate of 4.2 $\pm$ 0.8 nm/s, and relaxation at the trailing edge with an average rate of 3.8 $\pm$ 0.6 nm/s. The remaining two cells (out of eight) exerted pushing forces at the leading edge that deformed the vessels at an average rate of 1.2 $\pm$ 1.1 nm/s and relaxation at the trailing

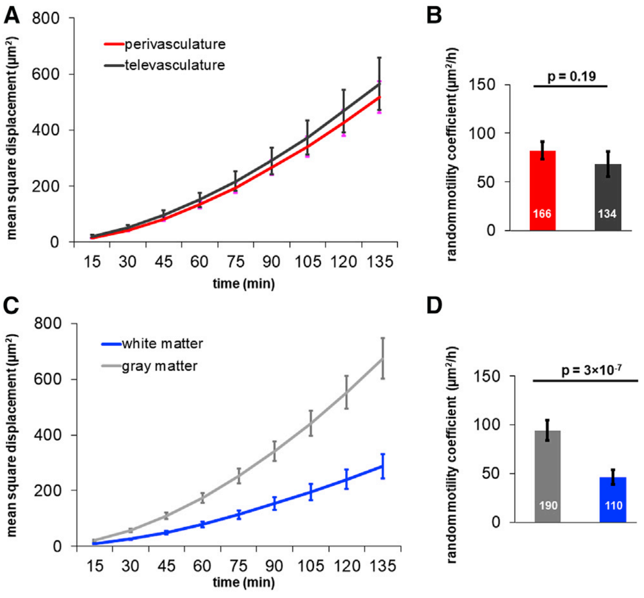

FIGURE 4 Cell motility is faster in gray matter than in white matter but is not influenced by the presence of vasculature. (*A*) The MSD plots of cells in the perivascular (56.3% of the cells) and televascular space (43.7% of the cells) are shown. (*B*) The random motility coefficient of cells in the perivascular and televascular space are not significantly different ($p = 0.19$). (*C*) The MSD plots of cells in gray matter and white matter are shown. (*D*) The random motility coefficient of cells in gray matter are higher than those in white matter ($p = 3 \times 10^{-7}$). Number of cells tracked are indicated in (*B*) and (*D*). Error bars represent SEM. To see this figure in color, go online.

edge with an average rate of 1.8 ± 0.9 nm/s. Thus, the brain-slice migration assay is able to potentially discriminate between models for cell migration in in vivo-like confined microenvironments.

Cell migration simulator predicts cell motility in the brain slice

The cell migration motility difference we observed in the brain-slice assay is summarized in Fig. 6 *A* as perivasculature in white matter, televasculature in white matter, perivasculature in gray matter, and televasculature in gray matter. The top panel shows wind rose plots of 20 randomly chosen cells in these categories. To understand the mechanistic basis of the differences, we utilized the two-dimensional cell migration simulator to predict cell migration behaviors for the four categories. Our data suggest stronger alignment between cell migration and vasculature compared with white matter tracts. So, we used CMS v1.0 with restrictions in one dimension to simulate the perivascular space. The stiffness of brain tissue is controversial (37). Numerous magnetic resonant elastography studies have shown that white matter is stiffer than gray matter in the human brain (38). However, other studies using atomic force microscopy indentation found gray matter tends to be stiffer than white matter (39), whereas nanoindentation experiments reported that white matter has higher stiffness (40). The range of shear stiffness for gray matter is 1.13–5.3 kPa and for white matter is 1.43–13.6 kPa from reported magnetic resonant elastography studies (38). In this study, we chose 10 pN/nm as the substrate spring constant for both gray and white matter because the modest stiffness differences between gray and white matter probably does not change cell motility (22). The difference of glioma migration rate could be regulated by signals from the local microenvironment because white matter shows an entirely different composition of matrix components (41). Therefore, we used modest changes in the number of clutches to model the modest difference between migration rates in gray matter ($N_c = 850$) and white matter ($N_c = 1000$). Fig. 6 *B* shows the simulated cell motility in these categories matching the behaviors observed in experiments. The top panel also shows wind rose plots of 12 randomly chosen cells in these categories. Simulated cell motility is significantly higher when $N_c = 850$ (gray matter) than $N_c = 1000$ (white matter) ($p = 7.4 \times 10^{-5}$), and not significantly different in 2D restricted (perivascular) and 2D free (televascular) space ($p = 0.72$). The simulation provides one possible explanation for the different cell migration motility in the four categories. Further insight into the mechanism will require further investigation that will require systematic perturbation of the motor-clutch-actin system, for example, as described in (23,31).

DISCUSSION

In this study, we presented quantitative tools to analyze the alignment of glioma cell migration with the pre-existing Scherer's structures inherent in brain tissue. The multimodal optical imaging approach allows us to assess the relative contributions of vasculature versus white matter tracts. We found U251 glioma cells in mouse brain slices do not follow white matter tracts. Rather, cells follow vasculature, even in white matter, although it does not affect their migration rate. Furthermore, cell motility is lower in white matter than in gray matter, an effect that is potentially explained by differential adhesivity. By imaging local vasculature deformation dynamics during cell migration, we found that U251 cells are capable of exerting traction forces that locally pull on their environment, suggesting the applicability of a "motor-clutch" model for migration.

Advanced optical imaging methods can provide new contrasts to study the migratory behavior of glioma cells in brain tissue dynamically. Although we used PS-OCT to visualize white matter tracts, other optical imaging modalities could potentially be used for this purpose as well. PS-OCT takes advantage of the birefringent nature of myelin sheath in the white matter. Other optical modalities have also demonstrated the capability of identifying white matter in the brain. For example, myelin is rich in lipids, and the vibrational contrast from the CH_2 bond can be

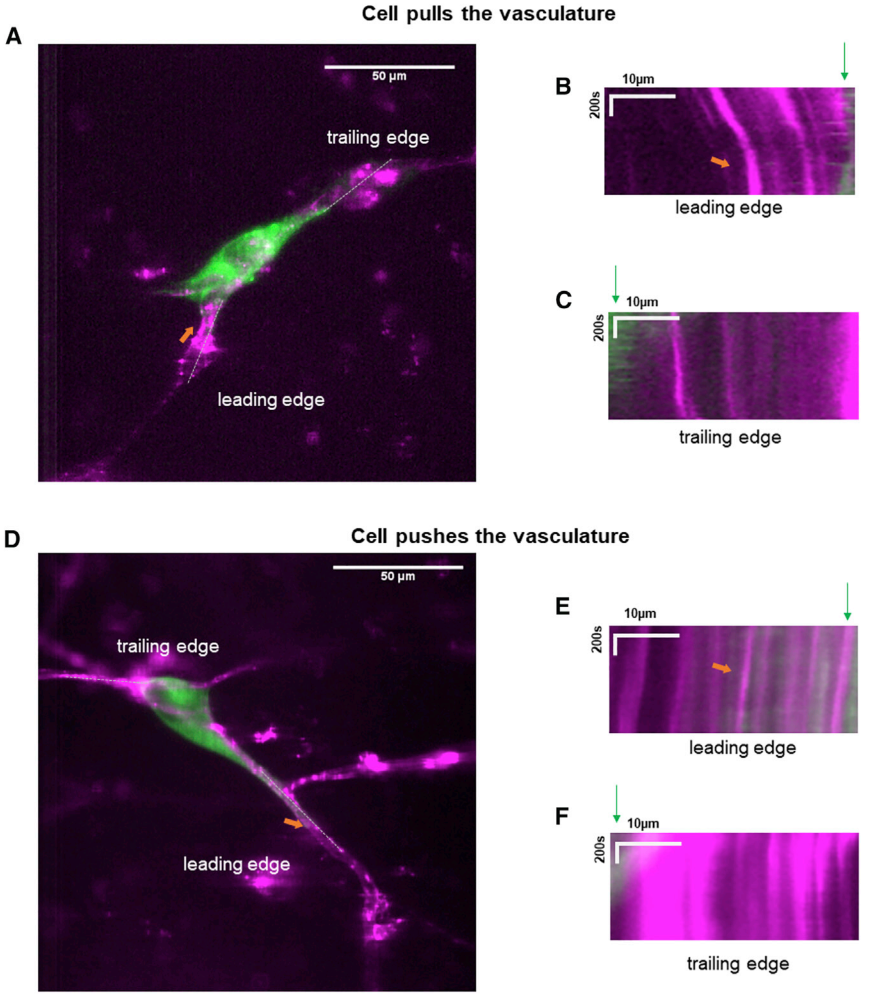

FIGURE 5 Dynamics of U251-cell-mediated mechanical deformation of local vasculature during migration. (*A*) A U251 cell (*green*) exerts force when it migrates along a blood vessel (*magenta*). (*B* and *C*) The kymographs indicate the cell applies load by pulling the blood vessel toward the cell body at the leading edge upon approach (*B*), whereas the blood vessel moves away from the cell at the trailing edge (*C*), presumably because of elastic recovery after cell deadhesion as the cell moves away. See Video S3 for the dynamics. (*D*) A U251 cell pushes the local blood vessel along which it migrates; the dynamics can be seen in the kymographs as the leading edge in (*E*) and trailing edge (*F*), as well as Video S4. Scale bars in (*A*) and (*D*): 50 μm. Scale bars in kymographs of (*B*), (*C*), (*E*), and (*F*): 10 μm in space and 200 s in time. To see this figure in color, go online.

highlighted by the chemically selective coherent anti-Stokes Raman scattering microscopy without exogenous labeling agents (42,43). Moreover, myelination is the dominant source of third harmonic generation contrast in the brain (15). These optical imaging modalities, combined with fluorescence-tagged cell imaging, provide other approaches to study the alignment between glioma cell migration and white matter tracts.

Despite our improved understanding of cancer progression and the development of many targeted therapies, patients with glioblastoma have seen little to no benefits. Many clinical trials have failed to slow disease progression and significantly improve clinical outcomes (44). By understanding the paths glioma cells use to invade the brain parenchyma, we could start to understand the mechanical and molecular mechanisms by which glioma cells invade healthy tissue and potentially identify druggable targets. Explanations of the fundamental physical phenomena underlying cell migration have emerged in recent years (45). However, the motility mechanisms actually used by tumor cells to migrate in vivo are still poorly understood.

Our study illustrates a method to study glioma dynamics in their native environment in detail. Using this method, we are able to obtain the first, to our knowledge, experimental evidence in brain tissue that cells use a motor-clutch mechanism to migrate in in vivo-like environments while they also potentially use other mechanisms to migrate, such as the osmotic engine model (36). In this respect, we were also able to provide one relatively simple possible explanation of the cell motility in the brain slice based on the cell migration simulator, in which modest changes in adhesivity to the environment mediate the modest difference in motility between gray and white matter. U251 cells, and perhaps glioblastoma cells generally, are able to navigate through diverse mechanical, chemical, and structural environments, implying that antimigratory strategies need to be robust across a wide range of in vivo structures and microenvironments to be effective. One direction of future study should be the investigation of the mechanism of cell migration in tissue and to what extent the motor-clutch and/or osmotic engine models apply to the 3D tissue microenvironment.

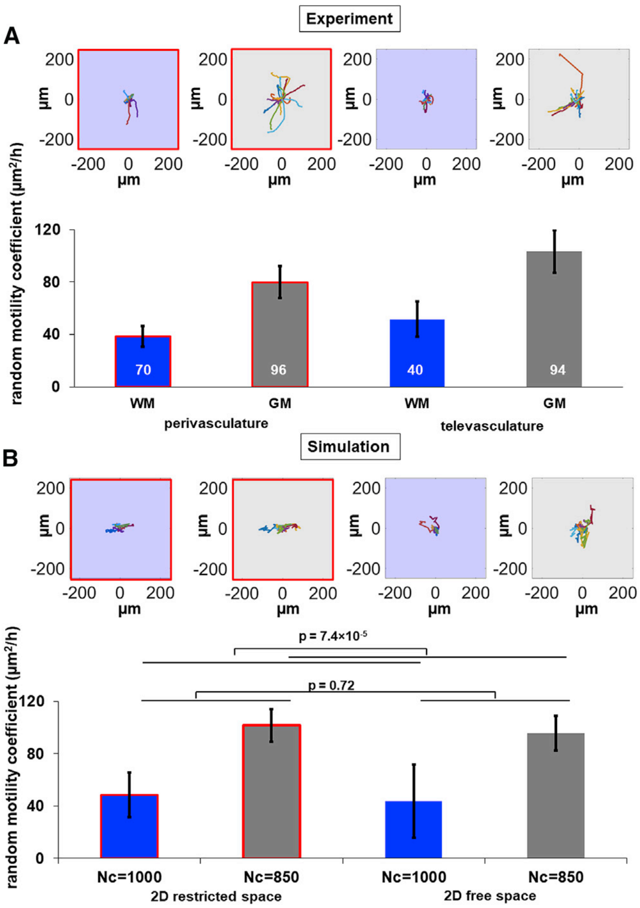

FIGURE 6 A motor-clutch-based model for cell migration can explain the modest differences in migration speed via modest differences in number of clutches. Cells were categorized as perivasculature in white matter, televasculature in white matter, perivasculature in gray matter, and televasculature in gray matter. The random motility coefficients of the cells are shown in each category for experiment (*A*) and simulation (*B*) from the cell migration simulator. Top panels in (*A*) and (*B*) show the wind rose plots of tracked cells and simulated cells. Simulated cell motility is significantly higher when $N_c = 850$ (*gray matter*) than $N_c = 1000$ (*white matter*) ($p = 7.4 \times 10^{-5}$), and not significantly different in 2D restricted (perivascular) and 2D free (televascular) space ($p = 0.72$). The number of cells tracked is indicated in (*A*) for experiments. The number of cells simulated in each category is 24. Error bars represent SEM. To see this figure in color, go online.

Despite the ability of U251 glioma cells to navigate diverse brain microenvironments, our results provide insight into the potential mechanisms of glioblastoma invasion into the brain. Compared with white matter tracts, vasculature is more correlated with glioma migration, suggesting that the perivascular space should be the primary target for therapy rather than white matter tracts. Because only one highly passaged cell line (U251) is used, more evidence is needed to confirm the weak alignment between glioma cell migration and white matter tracts as well as the lower cell motility in the white matter. To address the issue of generalizability to the clinic, low-passage patient-derived cell lines or primary cell lines should be studied in further work because highly passaged cell lines often fail to preserve key aspects of glioma biology (46).

SUPPORTING MATERIAL

Supporting Material can be found online at https://doi.org/10.1016/j.bpj.2019.08.010.

AUTHOR CONTRIBUTIONS

C.J.L. and D.J.O. designed the study. C.J.L. and G.A.S. performed brain-slice experiments. C.J.L. performed quantitative analysis of data and the simulations. C.J.L. and D.J.O. wrote the manuscript with input from all authors. D.J.O. oversaw the study.

ACKNOWLEDGMENTS

The authors thank Xiyao Jin for assistance with data collection and Aleksi Isomursu for help with the cell migration simulator.

The study was supported by research grants from National Institutes of Health (R01-CA-172986 and U54-CA-210190 to D.J.O.) and by the Graduate School Doctoral Dissertation Fellowship at the University of Minnesota (to C.J.L.). High-performance computing resources were provided by Minnesota Supercomputing Institute.

REFERENCES

1. Armento, A., J. Ehlers, …, U. Naumann. 2017. Chapter 5. Molecular mechanisms of glioma cell motility. *In* Glioblastoma. S. De Vleeschouwer, ed. Codon Publications, pp. 73–93.

2. Scherer, H. J. 1938. Structural development in gliomas. *Am. J. Cancer.* 34:333–351.

3. Cuddapah, V. A., S. Robel, …, H. Sontheimer. 2014. A neurocentric perspective on glioma invasion. *Nat. Rev. Neurosci.* 15:455–465.

4. Farin, A., S. O. Suzuki, …, P. Canoll. 2006. Transplanted glioma cells migrate and proliferate on host brain vasculature: a dynamic analysis. *Glia.* 53:799–808.

5. Hirata, E., H. Yukinaga, …, T. Okada. 2011. In vivo fluorescence resonance energy transfer imaging reveals differential activation of Rho-family GTPases in glioblastoma cell invasion. *J. Cell Sci.* 125:858–868.

6. Winkler, F., Y. Kienast, …, J. Herms. 2009. Imaging glioma cell invasion in vivo reveals mechanisms of dissemination and peritumoral angiogenesis. *Glia.* 57:1306–1315.

7. Watkins, S., S. Robel, …, H. Sontheimer. 2014. Disruption of astrocyte-vascular coupling and the blood-brain barrier by invading glioma cells. *Nat. Commun.* 5:4196.

8. Krusche, B., C. Ottone, …, S. Parrinello. 2016. EphrinB2 drives perivascular invasion and proliferation of glioblastoma stem-like cells. *eLife.* 5:e14845.

9. Wang, J., S. L. Xu, …, S. C. Yu. 2019. Invasion of white matter tracts by glioma stem cells is regulated by a NOTCH1-SOX2 positive-feedback loop. *Nat. Neurosci.* 22:91–105.

10. Claes, A., A. J. Idema, and P. Wesseling. 2007. Diffuse glioma growth: a guerilla war. *Acta Neuropathol.* 114:443–458.

11. Breckwoldt, M. O., J. Bode, …, B. Tews. 2019. Correlated MRI and ultramicroscopy (MR-UM) of brain tumors reveals vast heterogeneity of tumor infiltration and neoangiogenesis in preclinical models and human disease. *Front. Neurosci.* 12:1004.

12. Guillamo, J. S., F. Lisovoski, …, T. Lefrançois. 2001. Migration pathways of human glioblastoma cells xenografted into the immunosuppressed rat brain. *J. Neurooncol.* 52:205–215.

13. Beadle, C., M. C. Assanah, …, P. Canoll. 2008. The role of myosin II in glioma invasion of the brain. *Mol. Biol. Cell.* 19:3357–3368.

14. Giese, A., L. Kluwe, …, M. Westphal. 1996. Migration of human glioma cells on myelin. *Neurosurgery.* 38:755–764.

15. Farrar, M. J., F. W. Wise, …, C. B. Schaffer. 2011. In vivo imaging of myelin in the vertebrate central nervous system using third harmonic generation microscopy. *Biophys. J.* 100:1362–1371.

16. Wang, H., A. J. Black, …, T. Akkin. 2011. Reconstructing micrometer-scale fiber pathways in the brain: multi-contrast optical coherence tomography based tractography. *Neuroimage.* 58:984–992.

17. Li, T., C. J. Liu, and T. Akkin. 2019. Contrast-enhanced serial optical coherence scanner with deep learning network reveals vasculature and white matter organization of mouse brain. *Neurophotonics.* 6:035004.

18. Liu, C. J., O. Rainwater, …, T. Akkin. 2018. Polarization-sensitive optical coherence tomography reveals gray matter and white matter atrophy in SCA1 mouse models. *Neurobiol. Dis.* 116:69–77.

19. Ohnishi, T., H. Matsumura, …, T. Hayakawa. 1998. A novel model of glioma cell invasion using organotypic brain slice culture. *Cancer Res.* 58:2935–2940.

20. Valster, A., N. L. Tran, …, M. Symons. 2005. Cell migration and invasion assays. *Methods.* 37:208–215.

21. Ivkovic, S., C. Beadle, …, S. S. Rosenfeld. 2012. Direct inhibition of myosin II effectively blocks glioma invasion in the presence of multiple motogens. *Mol. Biol. Cell.* 23:533–542.

22. Klank, R. L., S. A. Decker Grunke, …, D. J. Odde. 2017. Biphasic dependence of glioma survival and cell migration on CD44 expression level. *Cell Rep.* 18:23–31.

23. Bangasser, B. L., G. A. Shamsan, …, D. J. Odde. 2017. Shifting the optimal stiffness for cell migration. *Nat. Commun.* 8:15313.

24. Wang, H., M. K. Al-Qaisi, and T. Akkin. 2010. Polarization-maintaining fiber based polarization-sensitive optical coherence tomography in spectral domain. *Opt. Lett.* 35:154–156.

25. Liu, C. J., A. J. Black, …, T. Akkin. 2016. Quantifying three-dimensional optic axis using polarization-sensitive optical coherence tomography. *J. Biomed. Opt.* 21:70501.

26. Liu, C. J., K. E. Williams, …, T. Akkin. 2017. Visualizing and mapping the cerebellum with serial optical coherence scanner. *Neurophotonics.* 4:011006.

27. Ernst, C., and B. R. Christie. 2006. Isolectin-IB 4 as a vascular stain for the study of adult neurogenesis. *J. Neurosci. Methods.* 150:138–142.

28. Chan, C. E., and D. J. Odde. 2008. Traction dynamics of filopodia on compliant substrates. *Science.* 322:1687–1691.

29. Bergman, A. J., and K. Zygourakis. 1999. Migration of lymphocytes on fibronectin-coated surfaces: temporal evolution of migratory parameters. *Biomaterials.* 20:2235–2244.

30. Estabridis, H. M., A. Jana, …, D. J. Odde. 2018. Cell migration in 1D and 2D nanofiber microenvironments. *Ann. Biomed. Eng.* 46:392–403.

31. Prahl, L. S., P. F. Bangasser, …, D. J. Odde. 2018. Microtubule-based control of motor-clutch system mechanics in glioma cell migration. *Cell Rep.* 25:2591–2604.e8.

32. Xiong, B., A. Li, …, H. Gong. 2017. Precise cerebral vascular atlas in stereotaxic coordinates of whole mouse brain. *Front. Neuroanat.* 11:128.

33. Frangi, A. F., W. J. Niessen, …, M. A. Viergever. 1998. Multiscale vessel enhancement filtering. *In* Medical Image Computing and Computer-Assisted Intervention — MICCAI'98. W. M. Wells, A. Colchester, and S. Delp, eds. Springer, pp. 130–137.

34. Wang, H., C. Lenglet, and T. Akkin. 2015. Structure tensor analysis of serial optical coherence scanner images for mapping fiber orientations and tractography in the brain. *J. Biomed. Opt.* 20:036003.

35. Ray, A., Z. M. Slama, …, P. P. Provenzano. 2017. Enhanced directional migration of cancer stem cells in 3D aligned collagen matrices. *Biophys. J.* 112:1023–1036.

36. Stroka, K. M., H. Jiang, …, K. Konstantopoulos. 2014. Water permeation drives tumor cell migration in confined microenvironments. *Cell.* 157:611–623.

37. Pogoda, K., L. Chin, …, P. A. Janmey. 2014. Compression stiffening of brain and its effect on mechanosensing by glioma cells. *New J. Phys.* 16:075002.

38. Hiscox, L. V., C. L. Johnson, …, N. Roberts. 2016. Magnetic resonance elastography (MRE) of the human brain: technique, findings and clinical applications. *Phys. Med. Biol.* 61:R401–R437.

39. Koser, D. E., E. Moeendarbary, …, K. Franze. 2015. CNS cell distribution and axon orientation determine local spinal cord mechanical properties. *Biophys. J.* 108:2137–2147.

40. Weickenmeier, J., R. de Rooij, …, E. Kuhl. 2016. Brain stiffness increases with myelin content. *Acta Biomater.* 42:265–272.

41. Giese, A., and M. Westphal. 1996. Glioma invasion in the central nervous system. *Neurosurgery.* 39:235–250, discussion 250–252.

42. Wang, H., Y. Fu, …, J. X. Cheng. 2005. Coherent anti-stokes Raman scattering imaging of axonal myelin in live spinal tissues. *Biophys. J.* 89:581–591.

43. Evans, C. L., X. Xu, …, G. S. Young. 2007. Chemically-selective imaging of brain structures with CARS microscopy. *Opt. Express.* 15:12076–12087.

44. Omuro, A., and L. M. DeAngelis. 2013. Glioblastoma and other malignant gliomas: a clinical review. *JAMA.* 310:1842–1850.

45. Petrie, R. J., H. M. Harlin, …, K. M. Yamada. 2017. Activating the nuclear piston mechanism of 3D migration in tumor cells. *J. Cell Biol.* 216:93–100.

46. Giannini, C., J. N. Sarkaria, …, C. D. James. 2005. Patient tumor EGFR and PDGFRA gene amplifications retained in an invasive intracranial xenograft model of glioblastoma multiforme. *Neuro-oncol.* 7:164–176.

Biophysical Journal, Volume *117*

Supplemental Information

Glioma Cell Migration Dynamics in Brain Tissue Assessed by Multimodal Optical Imaging

Chao J. Liu, Ghaidan A. Shamsan, Taner Akkin, and David J. Odde

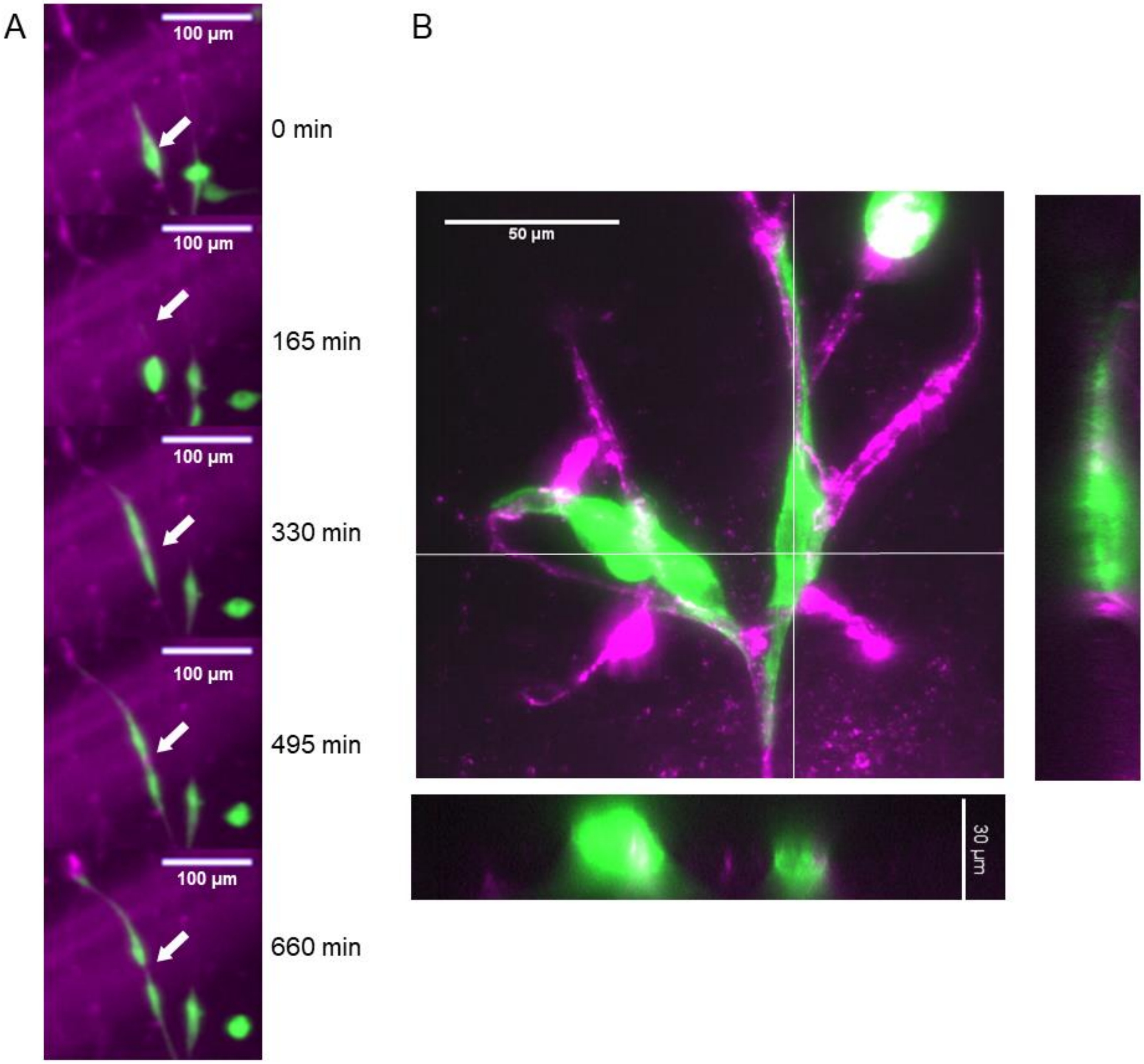

Supplementary Fig. 1 U251 cell migration dynamics in normal mouse brain slices.

(A) U251 cell division occurs in the perivascular space (indicated by the white arrows).
(B) Orthogonal views of perivascular U251 cell invasion in brain slice.

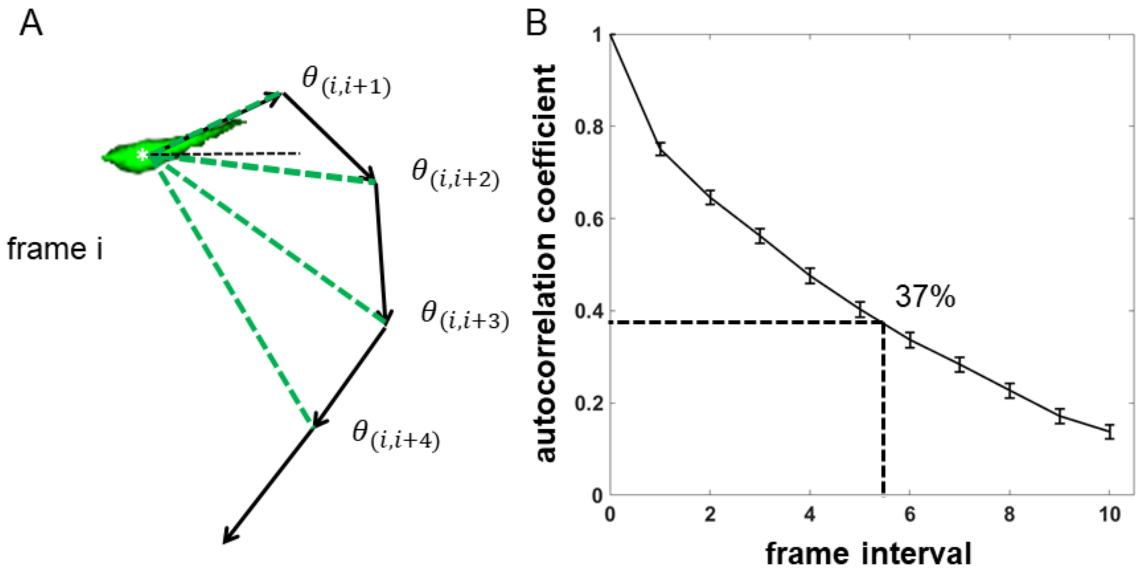

Supplementary Fig. 2. Calculation of cell migration angle

(A) Calculation of cell migration angles over different time intervals. (B) The autocorrelation coefficient of migration angles as a function of frame intervals (15 min/frame). The decay to 0.37 reflects the 1/e decay time. Measurements separated by 6 intervals were considered statistically uncorrelated.

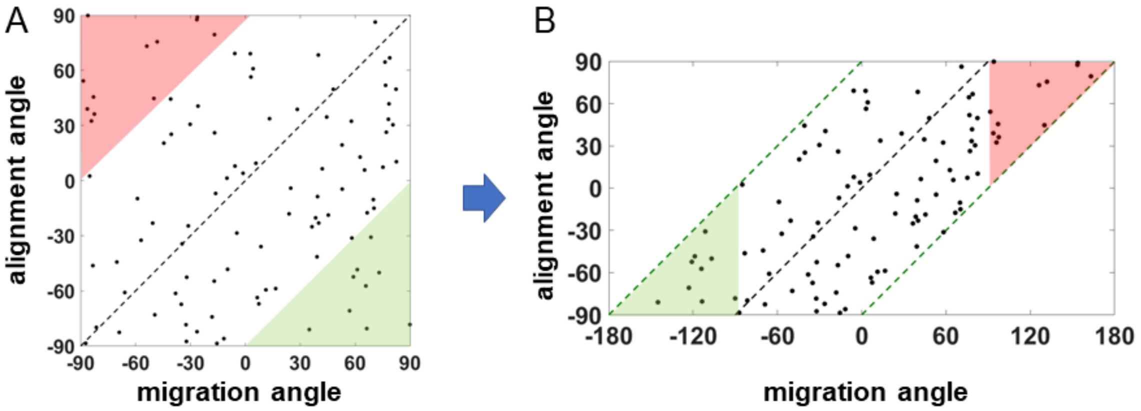

Supplementary Fig. 3 The analysis on the alignment of cell migration and local structure

100 simulated random walkers cells in the two shaded areas of Supplementary Fig. 3A are translated to the parallelogrammatic coordinate system (Supplementary Fig. 3B) separately. Supplementary Fig. 3B is used for the alignment index calculation of Fig. 3A.

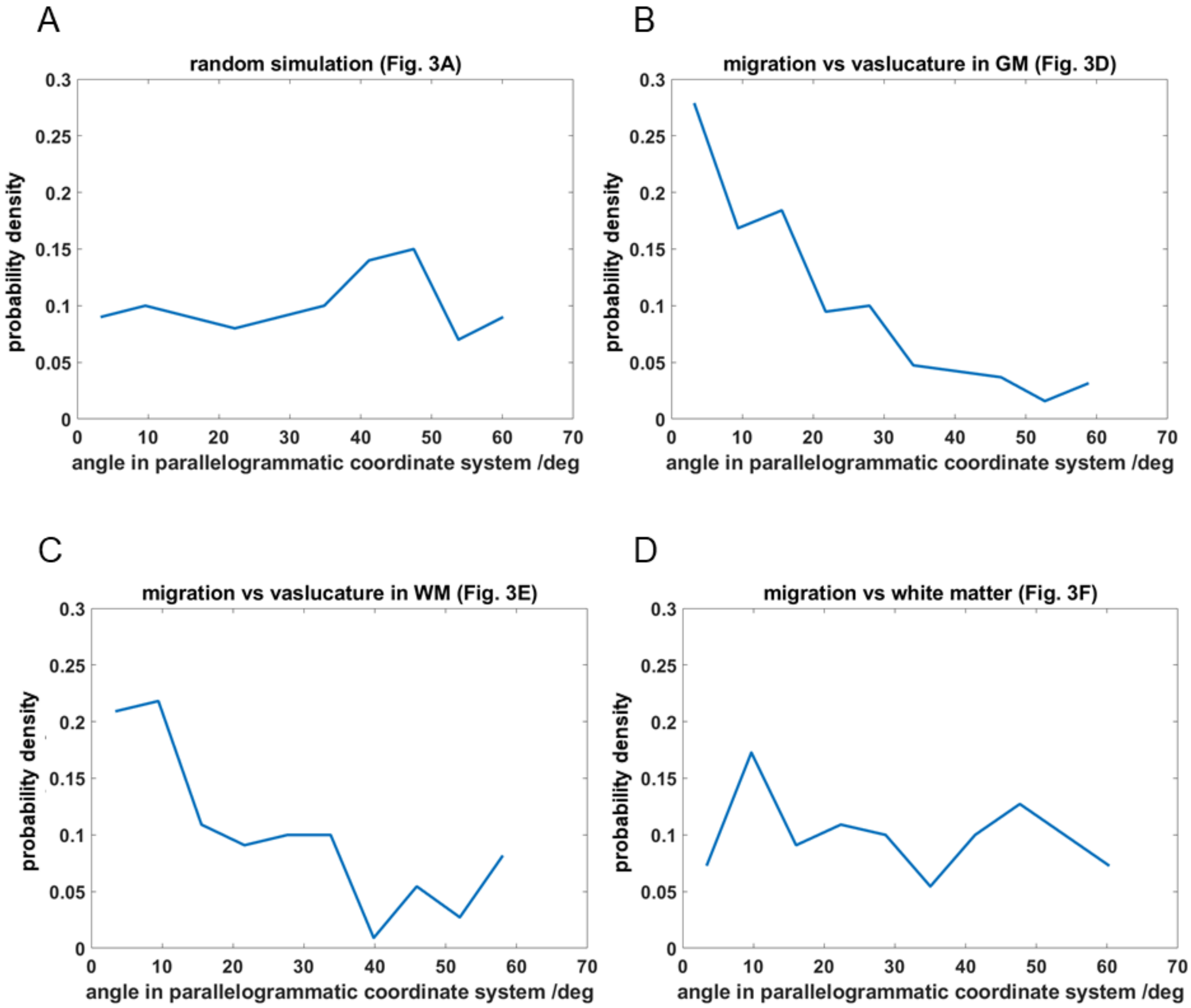

Supplementary Fig. 4 The probability density function of the scatter plots in Fig. 3

(A) The simulated $\phi = 0$ case. Cell migration angles are plotted versus the vessel orientations for cells in the gray matter (B) and white matter (C). (D) Cell migration angles are plotted against white matter tract orientations for cells in the white matter.

Supplementary videos

Supplementary video 1 (corresponding to Fig. 2A and 2B):

Cell migration along vasculature and the computational orientation maps.

Supplementary video 2 (corresponding to Fig. 2C and 2D):

Cell migration along white matter tracts and the computational orientation maps.

Supplementary video 3 (corresponding to Fig. 5A):

U251 cell deforms local vasculature during migration (vessel was pulled towards the cell at the leading edge).

Supplementary video 4 (corresponding to Fig. 5D):

U251 cell deforms local vasculature during migration (vessel was pushed away from the cell at the leading edge).
